# Pyocyanin reversibly inhibits *Chlamydia* by impairing newly formed elementary body infectivity and delaying the developmental cycle

**DOI:** 10.3389/fphar.2026.1921161

**Published:** 2026-09-11

**Authors:** Xinyang Sun, Wenjing Sang, Shunxin Xu, Leyi Li, Yu Zhao, Xiaofeng Bao

**Affiliations:** 1 School of Pharmacy, Nantong University, Nantong, China; 2 Jiangsu Key Laboratory of Inflammation and Molecular Targets, Nantong University, Nantong, China; 3 Nantong Key Laboratory of Small Molecular Drug Innovation, Nantong University, Nantong, China

**Keywords:** antichlamydial activity, *Chlamydia*, elementary body infectivity, intracellular developmental stage, pyocyanin

## Abstract

*Chlamydia* is an obligate intracellular bacterial pathogen responsible for sexually transmitted infections and blindness worldwide. In our search for antichlamydial agents from natural sources, we identified pyocyanin, a phenazine derivative from *Pseudomonas aeruginosa*. Previous studies showed that pyocyanin disables elementary body (EB) infectivity at low concentrations and inhibits intracellular growth at higher concentrations. In the present study, we show that its antichlamydial activity is primarily attributed to the intracellular developmental stage. Complete inhibition of detectable infectious progeny was achieved at 16 μM at the standard 36 hpi endpoint, while its effect on the infection stage reached only approximately 60% inhibition. Two independent delayed treatment assays revealed that pyocyanin reduces the infectivity of newly formed EBs without reducing their number. Furthermore, pyocyanin delays rather than arrests the chlamydial developmental cycle, and its inhibitory effect is reversible, requiring sustained drug exposure for optimal efficacy. These findings position pyocyanin among a growing class of antichlamydial agents that target distinct steps of the pathogen’s developmental cycle and support its potential as a chemical probe for chlamydial biology and a lead compound for drug development.

## 1 Introduction

*Chlamydiae* are Gram-negative obligate intracellular bacteria that cause a wide range of diseases in humans and animals. *Chlamydia trachomatis* is the leading bacterial sexually transmitted pathogen worldwide, with approximately 131 million new cases annually (Rowley et al., 2019). Untreated genital infections can lead to pelvic inflammatory disease, ectopic pregnancy, and infertility in women, and urethritis or epididymitis in men (Haggerty et al., 2010; Cunningham and Beagley, 2008). Additionally, *C. trachomatis* is the etiological agent of trachoma, the leading cause of preventable blindness in developing countries (Solomon et al., 2022). *Chlamydia pneumoniae*, another important human pathogen, causes respiratory tract infections such as bronchitis and pneumonia and has been implicated as a risk factor for atherosclerosis and Alzheimer’s disease (Balin et al., 2018; Campbell and Kuo, 2002). Current treatment for chlamydial infection relies primarily on antibiotic therapy, with doxycycline as the first-line regimen and azithromycin as an alternative (Kong and Hocking, 2015; Workowski et al., 2021; Centers for Disease Control and Prevention, 2024). Although these antibiotics are highly effective *in vitro*, concerns regarding their long-term use have emerged, including sporadic treatment failures, potential for antimicrobial resistance, antibiotic-induced persistent infection, and disruption of the host microbiota (Somani et al., 2000; Hogan et al., 2004; Bhengraj et al., 2010; Wyrick, 2010; Dethlefsen and Relman, 2011; Giacani et al., 2025; Wang et al., 2025). Given that no vaccine against chlamydial infection has yet been approved for human use, there is an urgent need to discover and develop novel antichlamydial agents with distinct mechanisms of action (Gottlieb et al., 2025; Pollak et al., 2026).

Chlamydiae possess a unique biphasic developmental cycle that alternates between two functionally and morphologically distinct forms: the infectious elementary body (EB) and the replicative reticulate body (RB). The cycle begins when an EB, a small (approximately 0.2–0.3 µm in diameter), spore-like particle, attaches to and enters a susceptible host cell through a pathogen-driven endocytic pathway. Once internalized, the EB resides within a membrane-bound vacuole, known as an inclusion, which is gradually modified by chlamydial effectors to become a protective replicative niche. Within this compartment, the EB differentiates into an RB, a larger (approximately 0.5–1.0 µm), metabolically active form that replicates by binary fission. Over time, RBs asynchronously convert back into infectious EBs, a process that involves condensation of the nucleoid and reorganization of the outer membrane. The newly formed EBs are then released from the host cell by either lysis of the host cell or extrusion of the entire inclusion, allowing them to initiate new rounds of infection (AbdelRahman and Belland, 2005; Elwell et al., 2016). This complex developmental cycle offers multiple intervention points for antichlamydial compounds. Recent efforts have identified numerous compounds that target distinct stages of the chlamydial developmental cycle. For instance, small molecule inhibitors targeting the type III secretion system (T3SS) have been shown to interfere with the translocation of effector proteins, thereby impairing chlamydial intracellular development and reducing the production of infectious progeny (Bailie et al., 2007; Muschiol et al., 2006). The kinase inhibitor H89 has been reported to slow inclusion growth and block RB-to-EB conversion by disrupting lysosomal cholesterol trafficking (Muñoz et al., 2021). In addition, compounds that interfere with host lipid metabolism and cholesterol acquisition, which are critical for chlamydial replication, have demonstrated potent antichlamydial activity (Heuer et al., 2009). Despite these advances, none of these compounds have proceeded from preclinical development to clinical evaluation, emphasizing the continued necessity to identify novel antichlamydial agents.

Natural products have long served as an important source of drug leads (Newman and Cragg, 2020; Li et al., 2022; Liu et al., 2024; Jin et al., 2025). Phenazines, a class of nitrogen-containing heterocycles isolated from both marine and terrestrial microorganisms, comprise over 100 naturally occurring derivatives and more than 6,000 synthetic analogues, exhibiting broad-spectrum pharmacological activities (Yan et al., 2021). Among them, pyocyanin (5-methyl-1-hydroxyphenazine) is a well-characterized virulence factor produced by *Pseudomonas aeruginosa*, known for its antibacterial and antifungal effects (Mudaliar et al., 2024). In our previous study, pyocyanin exhibited potent antichlamydial activity against multiple *Chlamydia* species, with an IC_50_ value of 0.02–0.03 μM against the production of infectious progeny EBs, comparable to that of tetracycline. Mechanistic investigations revealed that at low concentrations, pyocyanin impairs EB infectivity, thereby blocking the initial infection, whereas at higher concentrations, sustained drug exposure inhibits intracellular chlamydial growth (Li et al., 2018).

In the present study, we further investigated how pyocyanin inhibits chlamydial intracellular development. We provide evidence that pyocyanin reduces the infectivity of newly formed EBs within the inclusion without reducing the total number of EBs produced. The inhibitory effect is reversible upon compound withdrawal, and sustained drug exposure is required for optimal efficacy. Furthermore, pyocyanin delays rather than completely arrests the chlamydial developmental cycle, as inclusions that were initially suppressed can gradually develop upon extended incubation or after compound removal. These findings extend our understanding of pyocyanin’s antichlamydial activity and support its potential as a valuable chemical probe for studying chlamydial biology and as a lead compound for antichlamydial drug development.

## 2 Materials and methods

### 2.1 Strains, host cells and chemicals

*Chlamydia trachomatis* L2 (strain 434/Bu) and *Chlamydia muridarum* (strain Nigg II, mouse pneumonitis pathogen [MoPn]) were obtained from ATCC and propagated in HeLa cells. Cells were maintained in Dulbecco’s modified Eagle’s medium (DMEM) supplemented with 10% heat-inactivated fetal bovine serum (FBS) and 10 μg/mL gentamicin at 37 °C in a 5% CO_2_ atmosphere. A mouse polyclonal antiserum raised against *C. muridarum*, which cross-reacts with *C. trachomatis* L2, was produced in our laboratory as described previously (Bao et al., 2022) and used for immunostaining of both strains. FITC-conjugated goat anti-mouse secondary antibody was purchased from Sigma (F0257). Pyocyanin was obtained from Sigma (R9532, HPLC purity ≥98%) as a 5 mg/mL solution in dimethyl sulfoxide (DMSO), and stored at −20 °C in aliquots. Working solutions were prepared immediately before use.

### 2.2 *Chlamydia* infection and inhibition assay

The antichlamydial activity of pyocyanin was evaluated as previously described (Li et al., 2018). Briefly, HeLa cells seeded in 24-well plates were infected with *Chlamydia* at a multiplicity of infection (MOI) of 0.2 inclusion-forming units (IFU) per cell. Pyocyanin was added at the indicated time points, and 0.5% DMSO served as the negative control. Unless otherwise noted, cells were fixed with methanol at 36 h postinfection (hpi) and subjected to sequential staining with the primary antiserum and FITC-conjugated secondary antibody. Evans blue was used as a counterstain. Images were acquired using a Leica DMI3000B fluorescence microscope equipped with a ×20 objective, and inclusion number (each inclusion represents a single EB that has successfully entered a host cell and developed into a detectable inclusion, thereby reflecting the efficiency of chlamydial entry and subsequent intracellular establishment) and inclusion size (measured as the mean diameter of individual inclusions, which reflects the extent of inclusion growth and intracellular development) were measured using Image-Pro Plus 6.0 software. For quantification of infectious progeny EBs, infected cells were washed twice, scraped, and sonicated at 36 hpi to release EBs. The lysates were serially diluted 1:10 and used to reinfect fresh cells in 96-well plates, followed by immunofluorescence staining and inclusion counting as described above.

### 2.3 Cell counting Kit-8 (CCK8) assay

The cytotoxic effect of pyocyanin on host cells was assessed using the CCK-8 assay (Wang et al., 2022). Briefly, HeLa cells seeded in 96-well plates were cultured with 4, 8, 12, or 16 µM pyocyanin or 0.5% DMSO (vehicle control). Blank controls (medium without cells) were included in parallel. After 36, 48, 60, or 72 h of incubation, 10 µL of CCK-8 reagent (C0042, Beyotime) was added to each well, and the plates were incubated for an additional 1 h. Absorbance was then recorded at 450 nm. Cell viability was calculated as: viability (%) = [(OD_450_ of treated wells - OD_450_ of blank)/(OD_450_ of control wells - OD_450_ of blank)] × 100%.

### 2.4 Delayed treatment and withdrawal assay

HeLa cells seeded in 24-well plates were infected with *Chlamydia* at an MOI of 0.2. The infection process was synchronized by centrifugation at 1,000 rpm for 1 h at 4 °C (1 hpi was defined as the end of the centrifugation step). At 1 hpi, cells were washed three times to remove EBs that had not yet entered host cells (referred to as “non-internalized EBs” throughout this study). For the delayed treatment assay, 1 µM or 8 µM pyocyanin was added at indicated time points. For the withdrawal assay, pyocyanin was added at 1 hpi (immediately after the centrifugation step) and removed at indicated time points by washing three times, after which cells were further incubated in compound-free medium. For both assays, cells were either fixed for immunofluorescence staining or harvested at 36 hpi for quantification of infectious progeny EBs, as described above.

### 2.5 Preparation of genomic DNA for quantitative analyses

HeLa cells seeded in 6-well plates were infected with *Chlamydia* at an MOI of 0.2. At 2 hpi, cells were washed three times to remove non-internalized EBs. At 30 hpi, 8 µM pyocyanin was added, and cells were further cultured until 36 hpi. Infected cells were harvested using TRI reagent (T9424, Sigma-Aldrich) according to the manufacturer’s instructions, and genomic DNA was extracted from the interphase and organic phase. The relative genome copy numbers were determined by quantitative PCR (qPCR) targeting the *pdf* gene, using ChamQ Universal SYBR qPCR Master Mix (Q711-02, Vazyme) on a StepOnePlus RT-PCR system (Applied Biosystems, Carlsbad, CA, United States). The forward primer for the *pdf* gene was 5′-ctt​aga​tgg​gca​gca​gt-3′, and the reverse primer was 5′-ttg​ttt​tgt​cct​tgt​ctg​a-3'. The DMSO-treated sample at 30 hpi was set as the calibrator (relative genome copy number = 1), and the relative genome copy numbers of all other samples were normalized to this calibrator using the 2^−ΔCt^ method.

### 2.6 Long-term tolerance assay

HeLa cells seeded in 24-well plates were infected with *Chlamydia* at an MOI of 0.2. At 2 hpi, cells were washed three times to remove non-internalized EBs, followed by the addition of 4, 8,12, or 16 µM pyocyanin. Instead of the standard 36 h incubation, infected cells were cultured for extended periods up to 48, 60, or 72 h in the presence of pyocyanin. Inclusion number and size were analyzed by immunofluorescence staining. Infectious progeny EBs were quantified as described above, and the number of infectious progenies per inclusion was calculated based on titers and inclusion counts.

### 2.7 Recovery assay

HeLa cells seeded in 24-well plates were infected with *Chlamydia* at an MOI of 0.2. At 2 hpi, cells were washed three times to remove non-internalized EBs, and then cell were cultured until 12 hpi, followed by the addition of 4, 8,12, or 16 µM pyocyanin. At 36 hpi, the compound was removed by washing, and cells were further cultured for extended periods up to 48 or 60 h in the absence of pyocyanin. Inclusion number and size, as well as infectious progeny per inclusion, were analyzed as described in the long-term tolerance assay.

### 2.8 Transmission electron microscopy (TEM)

HeLa cells seeded in 24-well plates were infected with *C. trachomatis* L2 at an MOI of 0.2. At 2 hpi, non-internalized EBs were removed by three washes, and cells were cultured until 30 hpi. At 30 hpi, 8 µM pyocyanin was added, and incubation continued until 36 hpi. Cells were then fixed using the fixative provided by Wuhan Servicebio Technology Co., Ltd. (Wuhan, China) and shipped to the company for TEM analysis. Ultrathin sections were prepared, stained, and imaged by Servicebio. Representative micrographs taken at original magnifications of×1,500 and ×3,000 are shown, with EBs and RBs clearly indicated.

### 2.9 Statistical analysis

Data are presented as means ± standard deviations from three independent experiments and were analyzed by pairwise Student’s t tests. P < 0.05 and P < 0.01 are considered statistically significant and highly significant, respectively. Percent inhibition was calculated based on data in compound-treated samples relative to DMSO-treated control.

## 3 Results

### 3.1 Pyocyanin inhibits both the infection and intracellular developmental stages of *Chlamydia*

**FIGURE 1:**
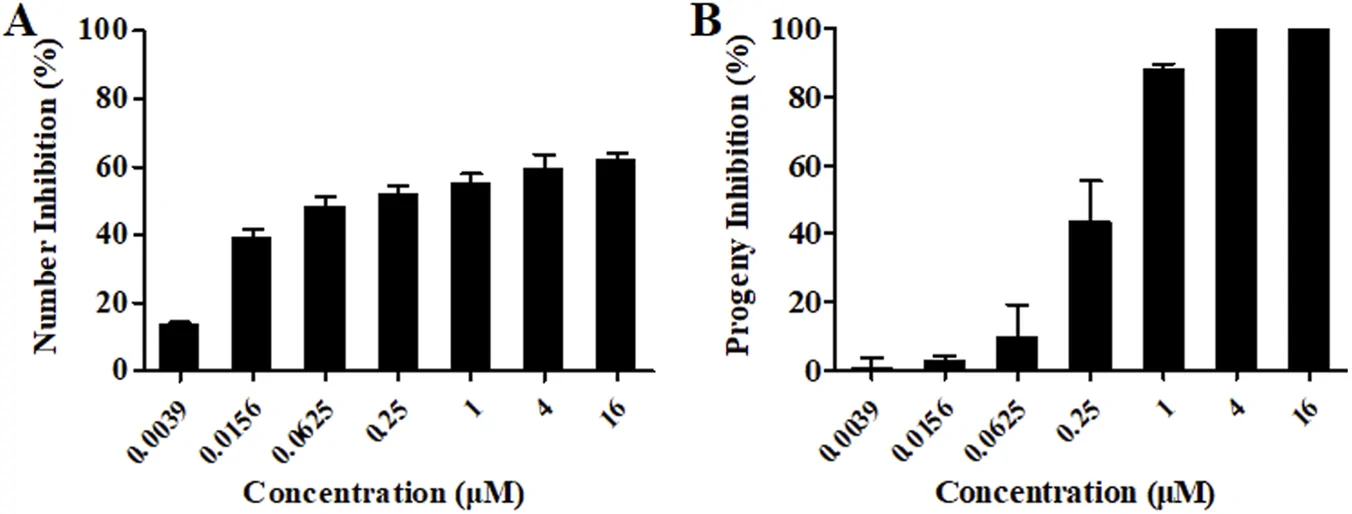
Inhibitory effects of pyocyanin on the infection and intracellular developmental stages of *C trachomatis* L2. HeLa cells infected with *Chlamydia trachomatis* L2 at an MOI of 0.2 were treated with various concentrations of pyocyanin. **(A)** For the infection-stage assessment, cells were exposed to pyocyanin at the time of infection. At 2 hpi, the compound and non-internalized EBs were removed by washing, and cells were further cultured until 36 hpi, followed by fixation and immunofluorescence staining to enumerate inclusion number. **(B)** For the intracellular developmental-stage assessment, non-internalized EBs were washed away at 2 hpi, followed by the addition of pyocyanin. Cells were cultured until 36 hpi and then harvested to quantify infectious progeny EBs. **(A,B)** 0.5% DMSO was used as a negative control, and percent inhibition was calculated relative to the DMSO-treated control. All data are presented as mean ± SD (n = 3).

Previous studies have revealed that pyocyanin inhibits both the infection and intracellular developmental stages of *Chlamydia* (Li et al., 2018). In the present study, when pyocyanin was administered only within the first 2 h postinfection (0–2 hpi, infection stage regimen), it reduced the number of inclusions (reflecting the quantity of EBs that successfully invaded host cells) at a concentration as low as 0.0039 µM. At the highest concentration tested (16 µM), which was the only concentration to cause host cell cytotoxicity (Li et al., 2018), the inhibition rate reached 62% (Figure 1A). Thus, although pyocyanin exhibits inhibitory activity against the infection process from very low concentrations, it does not achieve complete inhibition. In contrast, when pyocyanin was added from 2 to 36 hpi (2–36 hpi, intracellular developmental stage regimen), it completely suppressed chlamydial growth at 16 µM (Figure 1B), with a reduction in infectious progeny starting from 0.25 µM. These results indicate that pyocyanin exerts a stronger inhibitory effect on the intracellular developmental stage than on the infection stage, suggesting that this stage likely represents the primary target of its antichlamydial activity.

**FIGURE 2:**
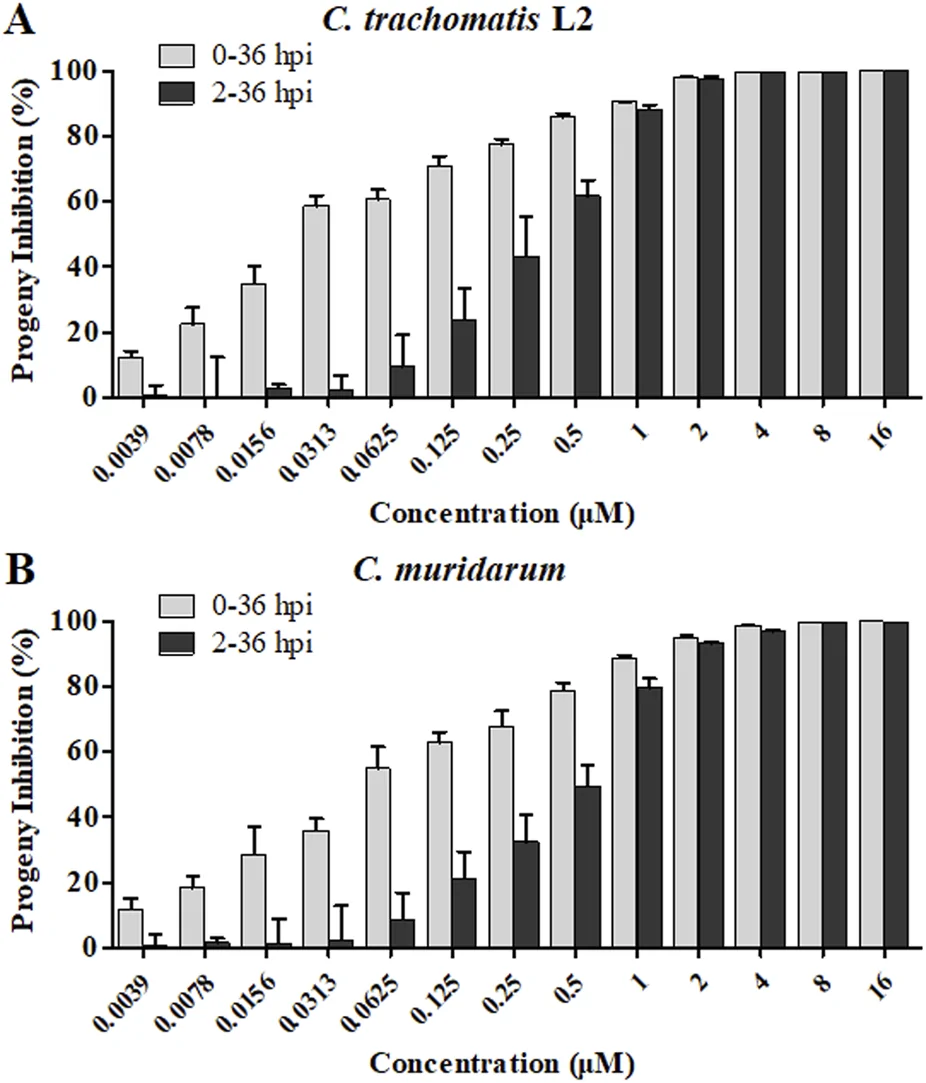
Inhibitory effects of pyocyanin on the full-cycle and the intracellular developmental stage of *Chlamydia*. HeLa cells infected with *Chlamydia trachomatis* L2 **(A)** or *C. muridarum* **(B)** at an MOI of 0.2 were treated with pyocyanin at the time of infection (0–36 hpi, full-cycle regimen) or starting at 2 hpi (2–36 hpi, intracellular developmental-stage regimen). At 36 hpi, cells were harvested to quantify infectious progeny EBs. 0.5% DMSO was used as a negative control, and percent inhibition was calculated relative to the DMSO-treated control. All data are presented as mean ± SD (n = 3).

Both the full-cycle (0–36 hpi) and intracellular developmental-stage regimens effectively inhibited chlamydial growth in a concentration-dependent manner, achieving complete inhibition at 16 µM (Figure 2A). Compared with the intracellular developmental-stage regimen, which began to reduce infectious progeny at 0.125 µM, the full-cycle regimen reduced the number of infectious progenies starting from as low as 0.0039 µM, and the magnitude of reduction was consistent with the decrease in inclusion numbers observed in the intracellular developmental-stage regimen (Figure 1A). This further indicates that low concentrations of pyocyanin affect only chlamydial infection. Moreover, the inhibitory effect of the full-cycle regimen was stronger than that of the intracellular developmental-stage regimen, and the difference gradually diminished with increasing pyocyanin concentrations, suggesting that the contribution of infection stage inhibition to the overall antichlamydial activity becomes less significant at higher concentrations.

Similar results were obtained in *C. muridarum* (Figure 2B). Pyocyanin inhibited chlamydial growth in a concentration-dependent manner under both the full-cycle and intracellular developmental-stage regimens, with complete inhibition achieved at 16 µM. The full-cycle regimen displayed greater inhibitory efficacy than the intracellular developmental-stage regimen at lower concentrations, whereas the difference between the two regimens diminished as pyocyanin concentrations increased. Collectively, these findings indicate that the antichlamydial activity of pyocyanin is mainly attributed to its action on the intracellular developmental stage.

### 3.2 Pyocyanin inhibition of *Chlamydial* intracellular development depends on treatment scheme

During the intracellular stages following EB entry, the chlamydial developmental cycle involves EB-to-RB differentiation, RB expansion by binary fission, and subsequent RB-to-EB redifferentiation (AbdelRahman and Belland, 2005; Elwell et al., 2016). To determine which stage(s) are primarily affected by pyocyanin, two different drug treatment schemes were applied. Because chlamydial infection is asynchronous, the infection process was synchronized by centrifugation at 1,000 rpm for 1 h at 4 °C, after which non-internalized EBs were removed by washing.

**FIGURE 3:**
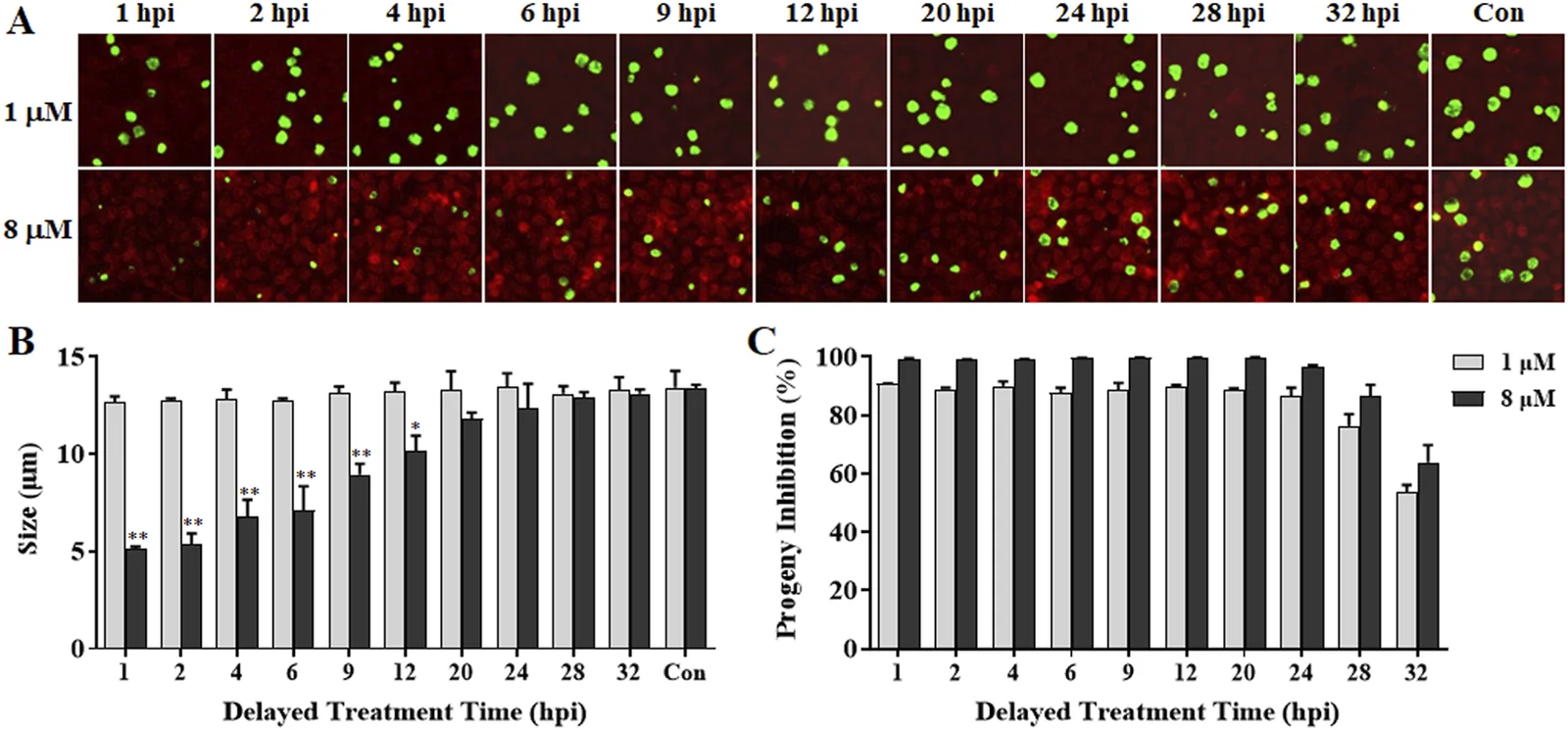
Delayed treatment assay. HeLa cells were infected with *Chlamydia trachomatis* L2 at an MOI of 0.2. The infection process was synchronized by centrifugation at 1,000 rpm for 1 h at 4 °C (1 hpi was defined as the end of the centrifugation step). At 1 hpi, non-internalized EBs were washed away, and 1 µM or 8 µM pyocyanin was added at indicated time points. At 36 hpi, cells were either fixed for immunofluorescence staining **(A,B)** or lysed for quantification of infectious progeny EBs **(C) (A)** Representative immunofluorescence images of inclusions stained with the primary antiserum (green), with host cells counterstained with Evans blue (red). **(B)** Inclusion size was measured using Image-Pro Plus 6.0 software. **(C)** Percent inhibition of infectious progeny EBs was calculated relative to the 0.5% DMSO-treated control. All data are presented as mean ± SD (n = 3). Statistical significance was determined by Student’s t‐test. *p < 0.05 and **p < 0.01 indicate significant differences compared with DMSO control.

For the delayed treatment assay, pyocyanin was added at different time points postinfection (Supplementary Figure S1). Addition of 1 µM pyocyanin at any time point did not cause any obvious change in inclusion size (Figures 3A,B). Inhibition of infectious progeny remained around 90% when pyocyanin was added before 24 hpi, but gradually diminished when added at or after 28 hpi, reaching only 54% at 32 hpi (Figure 3C). At 8 μM, earlier addition led to smaller inclusion size, and the progeny inhibition trend was similar to that at 1 µM but with stronger efficacy (Figure 3). These results indicate that although earlier addition of pyocyanin produces stronger inhibition, addition by 24 hpi is sufficient to maintain inhibition of infectious progeny, suggesting that sustained drug exposure from at least 24 hpi to 36 hpi is necessary for antichlamydial activity.

**FIGURE 4:**
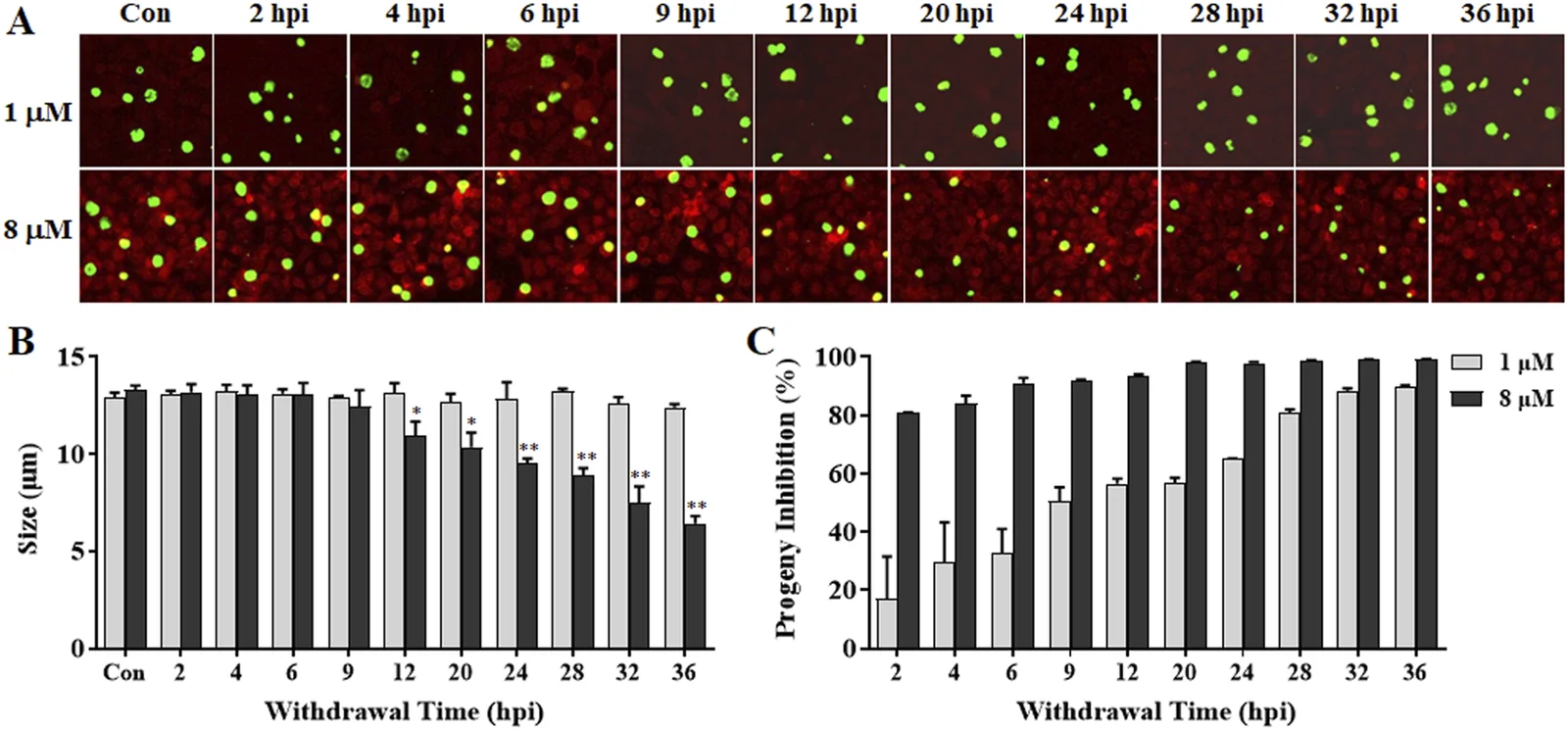
Withdrawal assay. HeLa cells were infected with *Chlamydia trachomatis* L2 at an MOI of 0.2. The infection process was synchronized by centrifugation at 1,000 rpm for 1 h at 4 °C (1 hpi was defined as the end of the centrifugation step). At 1 hpi, non-internalized EBs were washed away, and pyocyanin was added at 1 µM or 8 µM. At indicated time points, the compound was removed, and cells were further cultured until 36 hpi. Cells were then either fixed for immunofluorescence staining **(A,B)** or lysed for quantification of infectious progeny EBs **(C) (A)** Representative immunofluorescence images of inclusions stained with the primary antiserum (green), with host cells counterstained with Evans blue (red). **(B)** Inclusion size was measured using Image-Pro Plus 6.0 software. **(C)** Percent inhibition of infectious progeny EBs was calculated relative to the 0.5% DMSO-treated control. All data are presented as mean ± SD (n = 3). Statistical significance was determined by Student’s t-test. *p < 0.05 and **p < 0.01 indicate significant differences compared with DMSO control.

In the withdrawal assay, pyocyanin was added at 1 hpi and removed at various time points (Supplementary Figure S2). Consistent with the delayed treatment assay, at 1 μM, inclusion size remained unchanged regardless of removal time (Figures 4A,B), whereas inhibition of infectious progeny gradually increased with delayed drug removal, reaching 90% at 36 hpi (no withdrawal, positive control) (Figure 4C). At 8 μM, later removal resulted in smaller inclusions, and the progeny inhibition trend was similar to that at 1 µM but with greater potency (Figure 4). These results indicate that the later pyocyanin is removed, the stronger its inhibitory effect, further confirming that sustained drug exposure is required for its antichlamydial activity.

### 3.3 Pyocyanin reduces the infectivity of newly formed EBs within inclusions

As shown in the delayed treatment and withdrawal assays, different treatment schemes with 1 µM pyocyanin did not exhibit obvious inhibitory effects on inclusion size (Figures 3, 4) but strongly reduced infectious progeny EBs (Figures 3C, 4C). The reinfection assay, the most common method for evaluating antichlamydial activity, detects only infectious progeny rather than total progeny. Therefore, the reduction in titers could result either from a decrease in total progeny or from impaired infectivity of newly formed EBs. Given that pre-incubation of pyocyanin with EBs disables EB infectivity (Li et al., 2018), we hypothesized that at 1 μM, pyocyanin likely impairs the infectivity of newly formed EBs within inclusions, leading to reduced infectious progeny.

**FIGURE 5:**
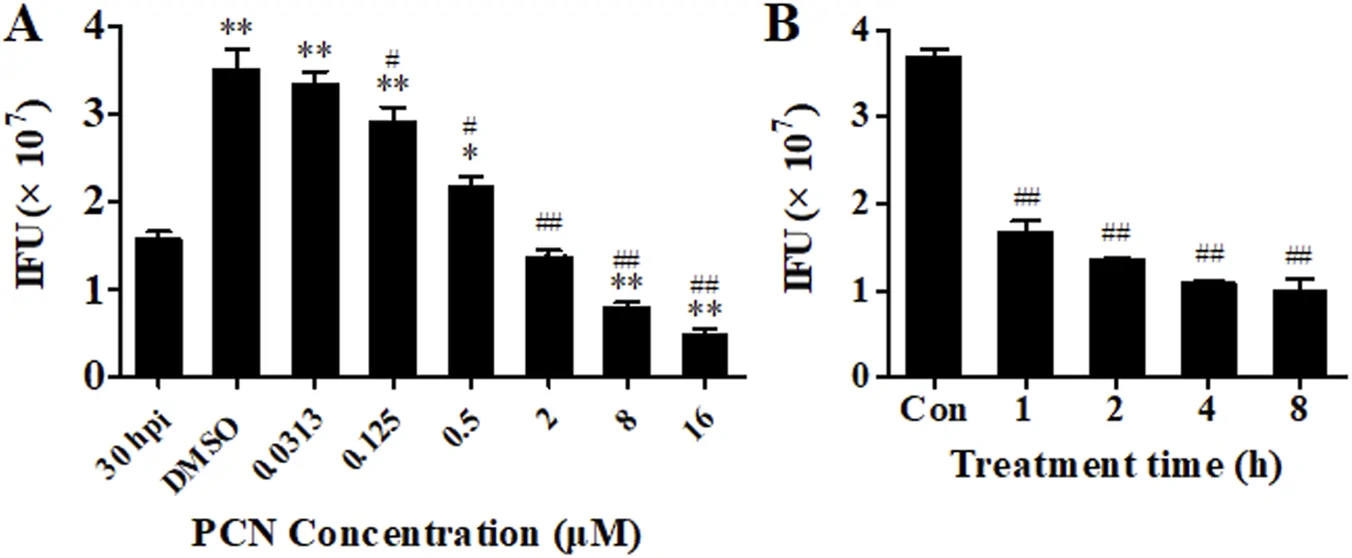
Pyocyanin reduces the infectivity of newly formed EBs within inclusions. HeLa cells were infected with *Chlamydia trachomatis* L2 at an MOI of 0.2. At 2 hpi, non-internalized EBs were washed away, and cells were further cultured. **(A)** At 30 hpi, various concentrations of pyocyanin were added. Cells were harvested at 36 hpi for quantification of infectious progeny EBs. The titer at 30 hpi served as the baseline control. **(B)** At 36 hpi, 8 µM pyocyanin was added. Cells were harvested after 1, 2, 4, or 8 h of incubation for quantification of infectious progeny EBs. All data are presented as mean ± SD (n = 3). Statistical significance was determined by Student’s t-test. *p < 0.05 and **p < 0.01 indicate significant differences compared with the 30 hpi DMSO control; #p < 0.05 and ##p < 0.01 indicate significant differences compared with the 36 hpi DMSO control.

To test this hypothesis, a delayed treatment assay was performed in which various concentrations of pyocyanin were added to infected cells at 30 hpi, and titers of infectious progeny EBs were measured at 36 hpi (Figure 5A). The results showed that EB titers increased from 1.57 × 10^7^/well at 30 hpi to 3.50 × 10^7^/well at 36 hpi in the DMSO control, indicating a significant increase in EB numbers during the 6 h proliferation period. Pyocyanin treatment reduced titers in a concentration-dependent manner. Notably, at 4 µM or higher, titers dropped to even lower than the level at 30 hpi. Since pre-existing EBs at 30 hpi cannot disappear, a likely explanation is that newly formed EBs lost their ability to reinfect host cells.

To verify whether pyocyanin affects the infectivity of newly formed EBs within inclusions, a second delayed treatment assay was conducted (Figure 5B). In this case, infected cells were cultured normally until 36 hpi to generate abundant infectious progeny EBs (titer: 3.50 × 10^7^/well), followed by the addition of 8 µM pyocyanin. After continued culture for 1–8 h, titers did not increase but rather decreased, with a further decreasing trend upon prolonged pyocyanin exposure. Moreover, after 4 h of continuous pyocyanin treatment, extending the duration did not further reduce titers, indicating that 4 h of incubation with 8 µM pyocyanin is sufficient to effectively disable the infectivity of newly formed EBs. Together, these results show that pyocyanin reduces the infectivity of newly formed EBs within inclusions.

**FIGURE 6:**
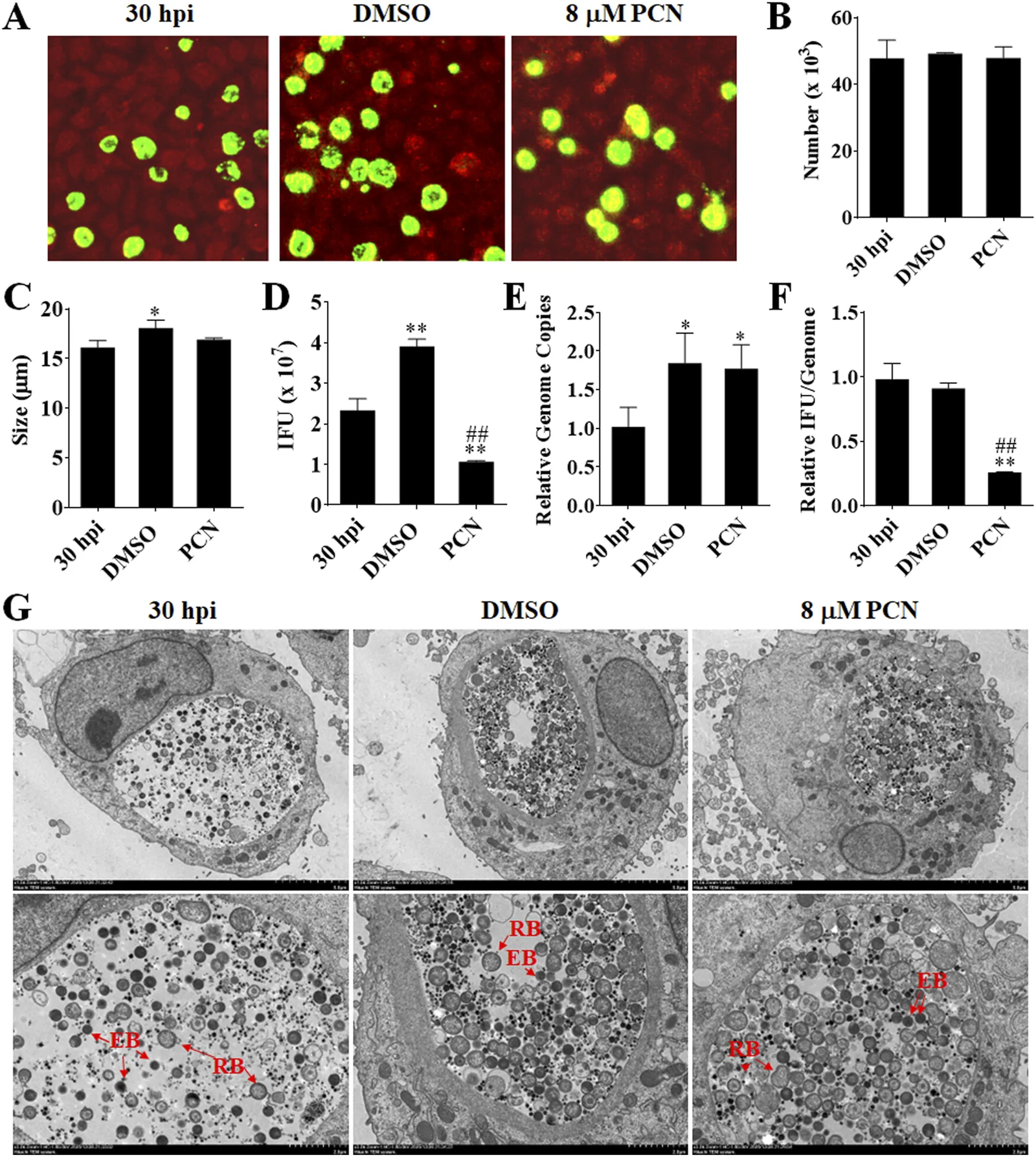
Pyocyanin does not reduce the number of pre-formed EBs within inclusions. HeLa cells were infected with *Chlamydia trachomatis* L2 at an MOI of 0.2. At 2 hpi, non-internalized EBs were washed away, and cells were further cultured. At 30 hpi, 8 µM pyocyanin (PCN) was added, and cells were further cultured until 36 hpi. Cells were then fixed for immunofluorescence staining **(A–C)** lysed for quantification of infectious progeny EBs **(D)** harvested for genomic DNA extraction **(E,F)** or processed for TEM analysis **(G) (A)** Representative immunofluorescence images of inclusions stained with the primary antiserum (green), with host cells counterstained with Evans blue (red). Inclusion number **(B)** and size **(C)** were measured using Image-Pro Plus 6.0 software. **(D)** Titers of infectious progeny EBs at 30 hpi and 36 hpi. Relative genome copy numbers **(E)** were determined by qPCR, and the relative IFU/genome ratio **(F)** was calculated. **(G)** Representative TEM micrographs at original magnifications of×1,500 and ×3,000, with EBs and RBs clearly indicated. DMSO-treated samples at 30 hpi and 36 hpi served as negative controls. All data are presented as mean ± SD (n = 3). Statistical significance was determined by Student’s t-test. *p < 0.05 and **p < 0.01 indicate significant differences compared with the 30 hpi DMSO control; #p < 0.05 and ##p < 0.01 indicate significant differences compared with the 36 hpi DMSO control.

To further confirm that pyocyanin impairs the infectivity of newly formed EBs without reducing their total number, infected cells were treated with 8 µM pyocyanin at 30 hpi and incubated until 36 hpi, followed by quantification of inclusion number, size, infectious progeny EB titers (Figures 6A–D), and relative genome copy numbers by qPCR (Figures 6E,F). Consistent with the results shown in Figure 5A, 8 µM pyocyanin reduced infectious progeny EB counts from 3.91 × 10^7^/well in the DMSO control to 1.06 × 10^7^/well (Figure 6D), whereas no significant effect on inclusion number or size was observed (Figures 6B,C). qPCR analysis revealed that relative genome copies increased 1.85-fold from 30 hpi to 36 hpi in the DMSO control, consistent with the increase in IFU titers during the same period. In contrast, pyocyanin treatment did not reduce genome copies compared with the DMSO control at 36 hpi (Figure 6E), indicating that total progeny production was not affected. The IFU-to-genome ratio remained comparable between the 30 and 36 hpi DMSO samples but was markedly lower in the pyocyanin-treated group at 36 hpi (Figure 6F). TEM analysis further visualized the effect of pyocyanin on EBs within inclusions (Figure 6G). At 36 hpi, abundant EBs were observed in both the DMSO control and pyocyanin-treated samples compared with the 30 hpi control. However, no apparent quantitative difference in EB numbers was observed between the DMSO and pyocyanin groups. These results further confirm that pyocyanin reduces the infectivity of newly formed EBs within inclusions but does not reduce the number of pre-formed EBs.

**FIGURE 7:**
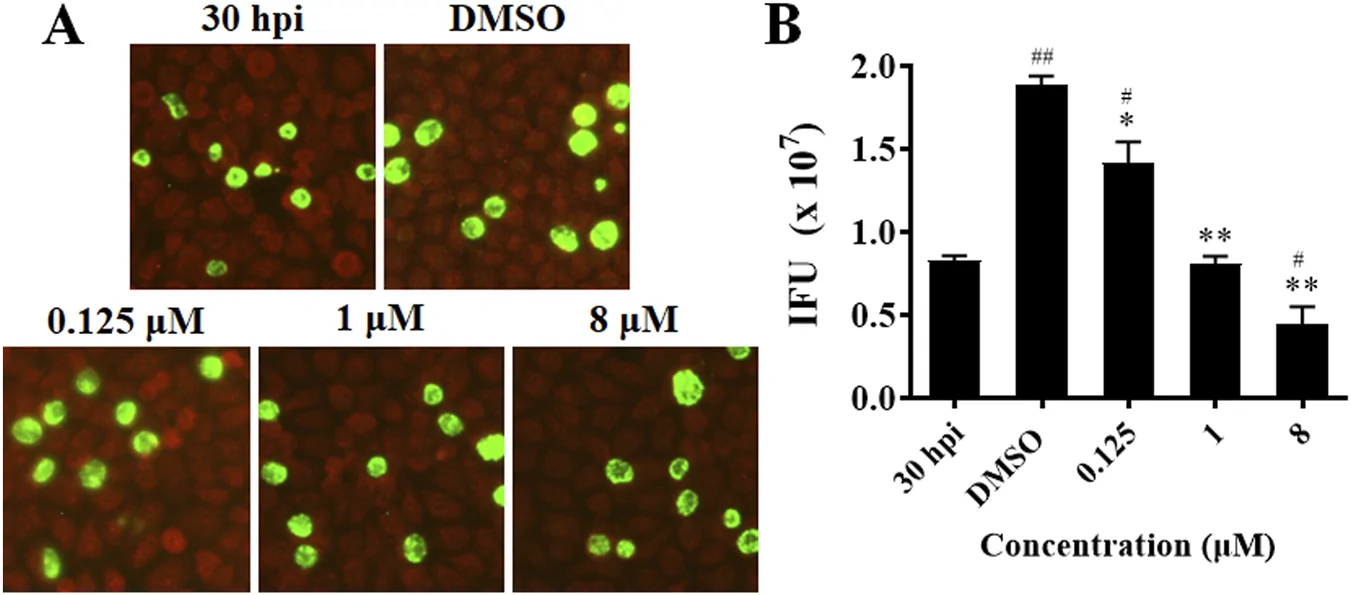
Pyocyanin reduces the infectivity of newly formed EBs within inclusions in *C. muridarum*. HeLa cells were infected with *C. muridarum* at an MOI of 0.2. At 2 hpi, non-internalized EBs were washed away, and cells were further cultured. At 30 hpi, 0.125, 1, or 8 µM pyocyanin were added, and cells were further cultured until 36 hpi. **(A)** Representative immunofluorescence images of inclusions stained with the primary antiserum (green), with host cells counterstained with Evans blue (red). **(B)** Titers of infectious progeny EBs at 30 hpi and 36 hpi. The titer at 30 hpi served as the baseline control. All data are presented as mean ± SD (n = 3). Statistical significance was determined by Student’s t-test. *p < 0.05 and **p < 0.01 indicate significant differences compared with the 30 hpi DMSO control; #p < 0.05 and ##p < 0.01 indicate significant differences compared with the 36 hpi DMSO control.

Similar results were obtained in *C. muridarum*. Immunofluorescence images (Figure 7A) showed no apparent differences in inclusion number or size between pyocyanin-treated and control groups. However, titration of infectious progeny (Figure 7B) revealed that pyocyanin treatment reduced the yield relative to the 36 hpi control. Notably, at 8 μM, the titer fell below the 30 hpi level, consistent with the impaired EB infectivity observed in *C. trachomatis* L2 (Figure 5A). These findings confirm that pyocyanin similarly compromises the infectivity of newly formed EBs in a different *Chlamydia* species.

### 3.4 Pyocyanin delays the *Chlamydial* developmental cycle

Although different treatment schemes with 1 µM pyocyanin had almost no effect on inclusion size, a higher concentration of 8 µM pyocyanin significantly and gradually reduced inclusion size with increasing treatment duration (Figures 3A, 4A). As inclusion size is closely associated with RB expansion, we hypothesized that this phenomenon results from pyocyanin delaying chlamydial developmental progression, particularly the RB expansion phase, leading to observed inclusion size reduction at the standard 36 hpi endpoint.

**FIGURE 8:**
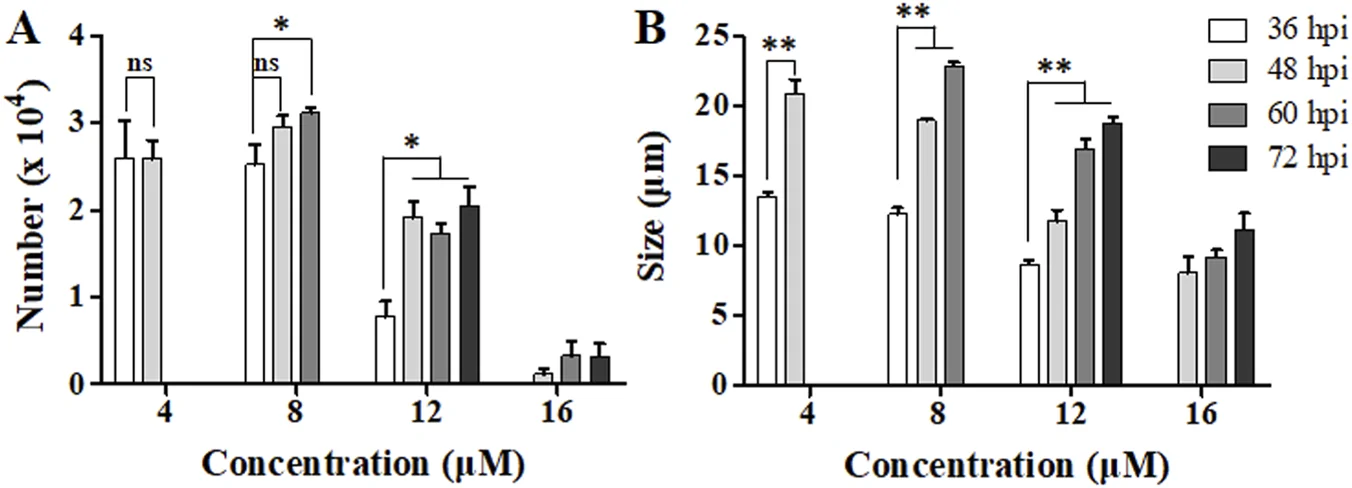
Pyocyanin delays the chlamydial developmental cycle. HeLa cells were infected with *Chlamydia trachomatis* L2 at an MOI of 0.2. At 2 hpi, non-internalized EBs were washed away, and various concentrations of pyocyanin were added. Cells were further cultured until 36, 48, 60, or 72 hpi, and then were fixed for immunofluorescence staining. Inclusion number **(A)** and size **(B)** were measured using Image-Pro Plus 6.0 software. All data are presented as mean ± SD (n = 3). Statistical significance was determined by Student’s t-test. *p < 0.05 and **p < 0.01 indicate significant differences compared with the DMSO control at the corresponding time point. NS, not significant.

To verify this hypothesis, a long-term tolerance assay was performed to observe whether inclusions could gradually enlarge when extending incubation to 48, 60, or 72 hpi with pyocyanin (Supplementary Figure S3). As shown in Figure 8A, at 12 µM pyocyanin, inclusion numbers obviously increased when detected at 48, 60, and 72 hpi. At 16 µM pyocyanin, although no inclusions were detected at 36 hpi, inclusions appeared when the life cycle was extended to 48 hpi or later, suggesting that inclusions initially suppressed to undetectable levels could gradually and slowly develop to become detectable upon extended incubation time. For 4 μM and 8 µM pyocyanin, due to their relatively lower concentrations and consequently weaker inhibitory effects, the number of inclusions suppressed to undetectable levels was small. Therefore, only a few inclusions became detectable again upon prolonged incubation time, resulting in a non-significant change in inclusion number. Notably, all inclusions at both concentrations had lysed by 72 hpi, and at 4 μM, lysis occurred as early as 60 hpi. Inclusion size data (Figure 8B) revealed that with prolonged incubation time, inclusion sizes increased significantly at all pyocyanin concentrations. Even at 16 μM, where no inclusions were detected at 36 hpi, inclusions of measurable size appeared by 48 hpi and continued to enlarge over time. Taken together, these results indicate that sustained pyocyanin treatment only suppresses chlamydial growth without achieving complete killing, thereby validating our hypothesis that pyocyanin exerts its antichlamydial activity by delaying the chlamydial developmental cycle.

### 3.5 Pyocyanin inhibits chlamydial growth in a reversible manner

**FIGURE 9:**
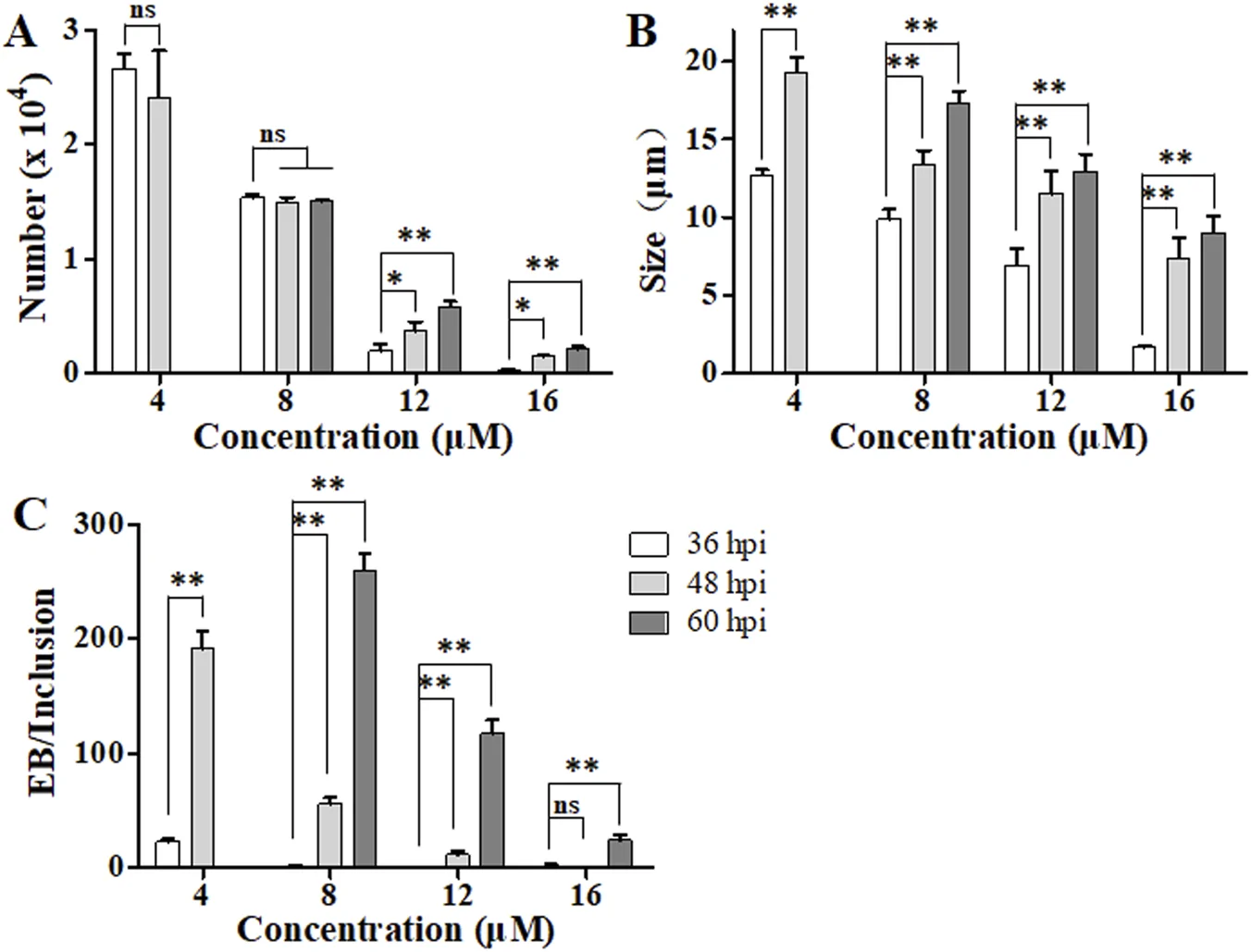
Pyocyanin inhibits chlamydial growth in a reversible manner. HeLa cells were infected with *Chlamydia trachomatis* L2 at an MOI of 0.2. At 2 hpi, non-internalized EBs were washed away, and cells were cultured until 12 hpi, followed by the addition of 4, 8,12, or 16 µM pyocyanin. At 36 hpi, the compound was removed, and cells were further cultured until 48 or 60 hpi in compound-free medium. Cells were then fixed for immunofluorescence staining. Inclusion number **(A)** size **(B)** and the number of infectious EBs per inclusion **(C)** were determined. All data are presented as mean ± SD (n = 3). Statistical significance was determined by Student’s t-test. *p < 0.05 and **p < 0.01 indicate significant differences compared with the DMSO control at the corresponding time point. NS, not significant.

To determine whether the antichlamydial activity of pyocyanin is reversible, a recovery assay was performed in which infected cells were treated with pyocyanin from 12 hpi to 36 hpi and then cultured in compound-free medium until 48 or 60 hpi (Supplementary Figure S4). When the detection time was extended beyond the standard 36 hpi endpoint in the absence of pyocyanin, both the number and size of inclusions increased with prolonged recovery time for all concentrations tested, except that the inclusion number at 4 and 8 µM showed no obvious change (Figures 9A,B). This indicates that suppressed inclusions resumed growth upon drug withdrawal. The lack of a marked recovery in inclusion number at 4 and 8 µM was likely due to the insufficient inhibitory potency at this concentration, as most inclusions had already developed to a detectable level. At 36 hpi, the number of infectious EBs per inclusion was nearly zero, but gradually increased with prolonged recovery time following compound removal (Figure 9C). These results collectively show that the inhibitory effect of pyocyanin on the *Chlamydia* life cycle can be reversed upon pyocyanin removal, indicating that pyocyanin exerts a reversible inhibitory effect on chlamydial growth.

### 3.6 Minimum drug exposure time required for optimal inhibition by pyocyanin

**FIGURE 10:**
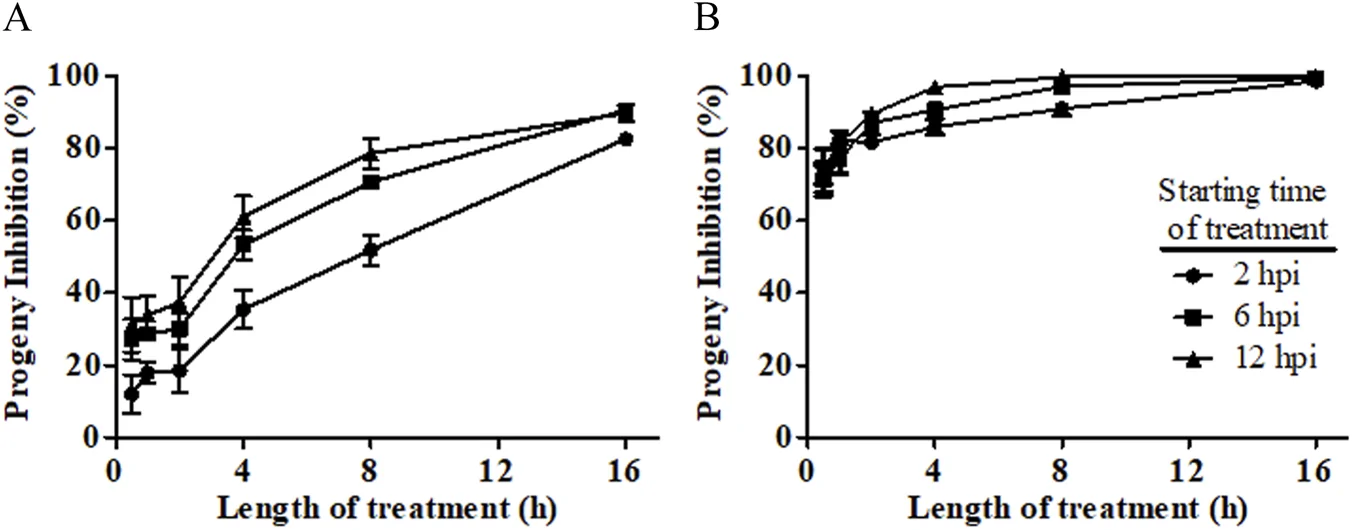
Minimum drug exposure time required for optimal inhibition by pyocyanin. HeLa cells were infected with *Chlamydia trachomatis* L2 at an MOI of 0.2. At 2 hpi, non-internalized EBs were washed away. At 2, 6, or 12 hpi, 1 µM **(A)** or 8 µM **(B)** pyocyanin was added. After incubation for 0.5, 1, 2, 4, 8, or 16 h, the compound was removed, and cells were further cultured until 36 hpi. Cells were then harvested for quantification of infectious progeny EBs. Percent inhibition was calculated relative to the 0.5% DMSO-treated control. All data are presented as mean ± SD (n = 3).

Given that pyocyanin inhibits chlamydial growth reversibly and requires sustained drug exposure, the question of the minimum exposure time required for optimal efficacy was addressed. Infected cells were treated with 8 µM pyocyanin at 2, 6, or 12 hpi (Figure 10B). After incubation for 0.5–16 h, the compound was removed, and cells were further cultured until 36 hpi. When pyocyanin was added at 2 hpi, at least 16 h of exposure was required to achieve near-maximal inhibition (99%). When addition was delayed to 6 or 12 hpi, the required exposure time was reduced to 8 h or 4 h, respectively, to achieve comparable inhibition. These data indicate that the later pyocyanin is added, the shorter the exposure time needed to reach maximal inhibition.

When a lower concentration of 1 µM pyocyanin was tested (Figure 10A), a similar trend was observed, albeit with weaker overall efficacy (maximum inhibition approximately 90%). At this concentration, addition at 6 or 12 hpi still required 16 h of exposure to achieve near-maximal inhibition, whereas addition at 2 hpi achieved only approximately 80% inhibition even with 16 h of exposure. This suggests that the minimum exposure time required for optimal efficacy depends on both the concentration and the duration of drug exposure. Collectively, treatment with 8 µM pyocyanin for 16 h is sufficient to achieve optimal inhibitory efficacy regardless of the time of addition, whereas lower concentrations or earlier addition times require longer exposure to reach maximal effect.

## 4 Discussion

Chlamydiae are common pathogens responsible for a wide range of diseases worldwide. Despite the favorable clinical efficacy of current antibiotic regimens, their use carries risks that can lead to treatment failure (Somani et al., 2000; Hogan et al., 2004; Bhengraj et al., 2010; Wyrick, 2010; Dethlefsen and Relman, 2011; Giacani et al., 2025; Wang et al., 2025). Given the absence of an approved vaccine for human use (Gottlieb et al., 2025; Pollak et al., 2026), novel antichlamydial agents are urgently needed. Natural products have served as a valuable source in this regard (Newman and Cragg, 2020; Li et al., 2022; Liu et al., 2024; Jin et al., 2025). A variety of such compounds, including plant phenolics, flavonoids, and alkaloids, have been shown to suppress chlamydial growth by interfering with host cell processes essential for bacterial replication (Li et al., 2017; Hanski and Vuorela, 2016; Lin et al., 2026). Our previous studies identified phenazines as possessing potent antichlamydial activity (Li et al., 2018; Bao et al., 2020; Bao et al., 2022). Specifically, pyocyanin exhibits a distinctive antichlamydial profile, characterized by its ability to both disable the infection process and inhibit the intracellular development.

A comparison of pyocyanin’s effects across different treatment regimens reveals that its antichlamydial activity is primarily attributed to the inhibition of the intracellular developmental stage, as complete inhibition was achieved at 16 μM, albeit with an onset concentration of 0.125–0.25 µM (Figures 1, 2). However, it is also important to note that pyocyanin reduces EB infectivity at concentrations as low as 0.0039 µM during the infection stage, despite reaching a maximum inhibition of only approximately 60% (Figure 1). This dual mode of action may be particularly advantageous, as the suppression of infectivity at extremely low concentrations lowers the number of *Chlamydia* that successfully establish infection, thereby lessening the burden on the intracellular-stage inhibition at higher concentrations. This cooperative mechanism appears to be a potent and efficient antichlamydial strategy. The kinase inhibitor H89 primarily affects RB replication and RB-to-EB conversion but does not reduce EB infectivity at similarly low concentrations (Muñoz et al., 2021). Likewise, inhibitors of T3SS have been reported to disrupt the chlamydial infectious cycle by blocking the translocation of effector proteins, thereby interfering with inclusion establishment and RB proliferation, without directly impairing EB infectivity (Muschiol et al., 2006; Bailie et al., 2007). In contrast, pyocyanin acts directly on both the EB and the RB, a distinctive feature that sets it apart from these agents and underlies its highly effective antichlamydial profile. Nevertheless, given that only inhibition of the intracellular developmental stage achieves complete suppression of detectable infectious progeny at the standard 36 hpi endpoint, future work should focus on elucidating the molecular basis by which pyocyanin disrupts this stage of the chlamydial life cycle.

A series of delayed treatment and withdrawal experiments revealed that at 8 μM, which achieves near-complete inhibition, both inclusion size and infectious progeny decreased with prolonged pyocyanin exposure. In contrast, at 1 μM, which produces a moderate effect, inclusion size remained largely unchanged regardless of treatment timing, whereas infectious progeny was markedly reduced (Figures 3, 4). This dissociation between inclusion size and infectious progeny at 1 μM suggested two possible explanations that pyocyanin might either reduce the total EB number within inclusions or impair the infectivity of a portion of EBs without affecting their total count. To distinguish between these possibilities, two independent delayed treatment assays were conducted. In the first assay, pyocyanin was added at 30 hpi and after 6 h of exposure at 4 μM or higher, infectious progeny titers fell below the level measured at 30 hpi (Figure 5A). In the second assay, pyocyanin was added at 36 hpi at a single concentration of 8 μM and within only 1 h, the titer decreased by more than 50% relative to the pre-addition level at 36 hpi (Figure 5B). In both cases, since pre-existing EBs cannot disappear, the decline in titer can only be attributed to the loss of infectivity of EBs within the inclusion. Importantly, several lines of evidence exclude the possibility that residual compound carryover or reduced EB production accounts for the observed titer reduction. First, the combined washing and dilution steps reduced any residual pyocyanin to far below 0.004 µM, a concentration at which pyocyanin inhibits inclusion formation by less than 15% (Figure 1A). Given that the reinfection assays showed more than 70% inhibition under pyocyanin treatment (Figures 5A, 6D), compound carryover can be ruled out as a confounding factor. Second, qPCR analysis revealed that pyocyanin did not reduce genome copies relative to the DMSO control (Figure 6E), indicating that total progeny production was unaffected. However, the IFU-to-genome ratio was markedly decreased following pyocyanin treatment (Figure 6F), suggesting that pyocyanin generates noninfectious EB-like particles. TEM further confirmed that pyocyanin did not reduce the number of EBs within inclusions, as no detectable change in the relative abundance of EBs and RBs was observed after pyocyanin treatment (Figure 6G). Collectively, these findings indicate that pyocyanin does not reduce EB numbers or block RB-to-EB conversion, but specifically impairs the infectivity of newly formed EBs. Taken together with our previous finding that pyocyanin inactivates extracellular EBs (Li et al., 2018), the present study shows that pyocyanin also inactivates newly formed intracellular EBs. Although its maximum efficacy is approximately 60% (Figure 1), this dual capacity complements the inhibitory effect on the intracellular developmental stage and contributes to the overall antichlamydial activity.

Our study also revealed that pyocyanin delays rather than completely arrests the chlamydial developmental cycle, and that this effect is reversible. When the detection time was extended beyond the standard 36 hpi in the continued presence of pyocyanin, both the number and size of inclusions continued to increase (Figure 8). Even at the highest concentration that achieved complete suppression at 36 hpi, detectable inclusions reappeared when detection was postponed to 48 hpi. Importantly, this reappearance was not due to host cell toxicity. At 4 and 8 μM, which already support the conclusions of developmental delay and reversibility, no cytotoxicity was detected over extended incubation (Supplementary Figure S5). The higher concentrations (12 and 16 µM) were included to visualize the delay more clearly, as they initially suppress inclusions to undetectable levels before reappearance upon extended incubation, but they are not essential for the core conclusions. Similarly, in a recovery experiment where pyocyanin was removed at 36 hpi, a comparable but slightly faster resumption of chlamydial growth was observed (Figure 9). These findings indicate that pyocyanin suppresses chlamydial growth by slowing the developmental cycle rather than by exerting a lethal effect, and that removal of the compound allows rapid recovery, underscoring the requirement for sustained drug presence. Consistent with this requirement, the inhibitory efficacy of pyocyanin was found to depend on the duration of drug exposure. At 8 μM, even a brief 0.5 h incubation significantly reduced infectious progeny, whereas near-complete inhibition required approximately 16 h of continuous exposure, regardless of when treatment was initiated (Figure 10B). A similar trend was observed at 1 μM, though longer incubation periods were needed to achieve comparable effects (Figure 10A). Notably, these exposure-response relationships were assessed at the standard 36 hpi endpoint. Given the delayed developmental progression observed in the long-term tolerance and recovery experiments, extending the detection time would likely diminish the apparent inhibition. Thus, while 16 h of drug exposure is sufficient to achieve maximal efficacy at the concentrations tested, sustained drug presence remains essential for maintaining the antichlamydial effect.

In summary, we characterized the antichlamydial effects of pyocyanin with a focus on the intracellular developmental stage. Our results demonstrate that pyocyanin exerts its primary inhibitory effect by targeting the replicative phase, specifically impairing the infectivity of newly formed EBs without reducing their total number, while requiring sustained drug exposure for optimal efficacy. These findings extend our previous work and place pyocyanin among a growing class of antichlamydial agents that target distinct steps of the chlamydial developmental cycle. Although pyocyanin itself is a *Pseudomonas aeruginosa* virulence factor with inherent cytotoxicity that limits its direct therapeutic application, its potent antichlamydial activity and distinctive dual mode of action support the phenazine scaffold as a valuable starting point for medicinal chemistry optimization.

## Data Availability

The raw data supporting the conclusions of this article will be made available by the authors, without undue reservation.
